# Antidiabetic Function of *Lactobacillus fermentum* MF423-Fermented Rice Bran and Its Effect on Gut Microbiota Structure in Type 2 Diabetic Mice

**DOI:** 10.3389/fmicb.2021.682290

**Published:** 2021-06-24

**Authors:** Xiaojuan Ai, Cuiling Wu, Tingting Yin, Olena Zhur, Congling Liu, Xiaotao Yan, CuiPing Yi, Dan Liu, Linhu Xiao, Wenkai Li, Binbin Xie, Hailun He

**Affiliations:** ^1^School of Life Sciences, Central South University, Changsha, China; ^2^Department of Biochemistry, Changzhi Medical College, Changzhi, China; ^3^School of Chemistry and Biology Engineering, Changsha University of Science and Technology, Changsha, China; ^4^Department of Biochemistry and Molecular Biology, School of Preclinical Medicine, Guangxi Medical University, Nanning, China; ^5^Microbial Technology Institute and State Key Laboratory of Microbial Technology, Shandong University, Qingdao, China

**Keywords:** rice bran, *Lactobacillus fermentum* MF423, antidiabetic, gut microbiota, antioxidant activity

## Abstract

Rice bran is an industrial byproduct that exerts several bioactivities despite its limited bioavailability. In this study, rice bran fermented with *Lactobacillus fermentum* MF423 (FLRB) had enhanced antidiabetic effects both *in vitro* and *in vivo*. FLRB could increase glucose consumption and decrease lipid accumulation in insulin resistant HepG2 cells. Eight weeks of FLRB treatment significantly reduced the levels of blood glucose and lipids and elevated antioxidant activity in type 2 diabetic mellitus (T2DM) mice. H&E staining revealed alleviation of overt lesions in the livers of FLRB-treated mice. Moreover, high-throughput sequencing showed notable variation in the composition of gut microbiota in FLRB-treated mice, especially for short-chain fatty acids (SCFAs)-producing bacteria such as *Dubosiella* and *Lactobacillus*. In conclusion, our results suggested that rice bran fermentation products can modulate the intestinal microbiota and improve T2DM-related biochemical abnormalities, so they can be applied as potential probiotics or dietary supplements.

## Introduction

Rice (*Oryza sativa*), a staple cereal grain, is consumed by 3.5 billion people worldwide (Wu et al., 2020). Rice bran is a byproduct of the rice milling process and contains diverse ingredients (dietary fiber, proteins, unsaturated fatty acids phenolics and γ-oryzanol), which exhibit a wide range of bioactivities, such as anti-hyperglycemic, anti-inflammatory, anti-dyslipidemia and antioxidant activity against type 2 diabetes mellitus (T2DM) (Sivamaruthi et al., 2018). Unfortunately, although they have outstanding bioactivity benefits and excellent application prospects, most parts of rice bran are not fully utilized and are directly discarded or used as animal feed, which leads to great waste. Thus, it is urgent to develop deep processing approaches for rice bran to improve its value.

Emerging evidence suggests that microbial fermentation can create high-value products that show multifarious biological effects. Punia et al. reported that rice bran fermentation with *Aspergillus oryzae* could enhance bioactive compounds and antioxidant potential (Punia et al., 2021). Fungi fermentation of rice bran could increase the bioactive composition and anti-inflammatory functions (Islam et al., 2017). *Lactobacilli*, a kind of probiotic, are considered safe and nutritional supplements in food processing. In addition, *Lactobacilli* exhibit several health benefits, such as decreasing the levels of glucose and lipids and improving oxidative stress (Giraffa, 2014; Slattery et al., 2019). A previous study reported that *Lactobacillus mali* APS1 accelerated weight loss and ameliorated lipid metabolism and glucose homeostasis by regulating SIRT-1/Nrf-2 and gut microbiota in rats (Chen et al., 2018). *Lactobacillus plantarum* ATCC14917 was used to ferment apple juice and the results demonstrated that it possessed high antioxidant activity by increasing 2,2-diphenyl-1-picrylhydrazyl (DPPH) radical-scavenging activity (Li et al., 2019). Our previous study also found that rice bran produced by *Lactobacilli* fermentation could improve the flavor of the product, and the fermentation product had good antioxidant activity and antiaging effects (Wang M. et al., 2020). These studies indicated that *Lactobacilli* fermentation products could further enrich the bioactivities of fermentation substrates. Recently, a large number of studies have found that using *Lactobacillus* to ferment cereal crops can significantly increase the quantity and kinds of bioactivity compounds of *Lactobacillus* fermented products. The soluble β-glucan is a kind of dietary fiber, which can obviously reduce the cholesterol level in T2DM. Luana et al. found that the content of soluble β-glucan was 40% higher in the *Lactobacillus* fermented oat extracts than that of in the unfermented extracts (Luana et al., 2014). The amount of free phenolic acids was significantly increased in whole grain barley extracts fermented with *Lactobacillus* (Hole et al., 2012), which were verified to inhibit adipogenesis and obesity (Seo et al., 2015). Similarly, Zhang J. Y. et al. (2018) found that fermented barley extracts with *Lactobacillus plantarum* dy-1 rich in vanillic acid increased glucose consumption and reduced proinflammatory cytokine secretion in HepG2 cells. Furthermore, a metabolomics assay was utilized to investigate the changes of components in barley during fermentation with *Lactobacillus plantarum* dy-1 through ultra-high performance liquid chromatography tandem with high resolution mass spectrometry (UPLC-HRMS). The results showed that the abundance of indole-3-lactic acid was significantly increased in barley fermentation extracts (Zhao et al., 2021), which was reported lower in T2DM subjects than that of in control subjects (Jennis et al., 2017). Another study found that the indole-3-lactic acid was considered as a crucial metabolite produced via transforming tryptophan with *lactobacillus* fermentation, which was identified to maintain the gut homeostasis to improve insulin resistance through decreasing the intestinal permeability and circulating lipopolysaccharide levels in high fat diet treated mice (Galligan, 2018). Herein, we hypothesize that the fermentation products which were obtained from the fermentation of rice bran with *Lactobacilli* may have diverse biological activities.

Clinically, approximately 90% of diabetes patients have been diagnosed with T2DM, which is characterized by a reduction in insulin sensitivity and dysfunction of β-cells with hyperglycemia and dyslipidemia (Zhang et al., 2016; Punthakee et al., 2018). Recently, growing evidence has verified that the gut microbiota is very closely related to the pathophysiology of T2DM (Dao and Clément, 2018). Dysbiosis of gut microbiota may lead to the disruption in metabolism, such as dyslipidemia and insulin resistant (Chen and Devaraj, 2018). Notably, significant differences in gut microbiota, including composition, richness, diversity and function, are observed between the healthy population and diabetic patients (Qin et al., 2012). Many converging lines of research indicate that the number of probiotics and opportunistic pathogens shows opposite tendency in T2DM (Qin et al., 2012; Chang et al., 2017). A study shows that bacteria belong to the phylum Firmicutes and the class Clostridia has lower amount in T2DM patients (Larsen et al., 2010). Tabusi et al. (2021) found that the abundance of *Lactobacillus* decreased in gut microbiota of db/db mice. While several opportunistic pathogens, such as *Bacteroides caccae, Clostridium ramosum, and Eggerthella lenta* increased significantly in T2DM (Woo et al., 2004). Functionally, the relevant metabolic pathways of sulfate reduction and oxidative stress resistance were enhanced (Zhu et al., 2020). Intriguingly, animal experiments showed that fecal transplantation from normal mice to high-fat/high-sugar-fed mice can reverse hyperglycemia and insulin resistance (Sung et al., 2017). Therefore, these results suggested that the gut microbiota played key roles in the development of T2DM. Short-chain fatty acid (SCFA), generated from undigested carbohydrates by gut microbiota, strengthen gut barrier function by supporting energy for epithelial growth and resisting invading microbes (Wang Y. X. et al., 2020; Liu et al., 2021). Moreover, SCFAs can regulate the relative expression of proteins controlling satiety and then reduce appetite to improve T2DM (Kim et al., 2018).

In the present study, *Lactobacillus fermentum* MF423, a probiotic separated from Chinese rice noodle wastewater, was selected to ferment rice bran and investigate the biological effect of the fermentation product (FLRB). The cell viability assay of FLRB was revealed by using 3-(4,5-dimethylthiazol-2-yl)-2,5-diphenyltetrazolium bromide (MTT). The antidiabetic effects of FLRB both *in vitro* and *in vivo* were determined. *In vitro*, FLRB-treated insulin-resistant HepG2 cells were used to evaluate the effects of improving lipid metabolism and glucose consumption. *In vivo*, insulin-resistant mice induced by a high-fat diet were utilized to assess the effects of ameliorating blood glucose and lipid accumulation and antioxidant activity. In addition, the modulation effects of gut microbiota in mice by 16S rRNA gene sequencing and bioinformatics analysis were investigated. Finally, the regulation of SCFA levels was analyzed by gas chromatography-mass spectrometry (GC-MS) analysis. The results of this study may lay the foundation for the comprehensive utilization of rice bran and suggest the potential value of rice bran fermentation products for the treatment of T2DM.

## Materials and Methods

### Reagents and Materials

*L. fermentum* MF423 was identified by 16S rRNA gene sequencing and kept at −80°C before use. The fresh rice bran was purchased from Chongqing, China. The rice bran was obtained from rice milling process by the McGill mill as described by Rebecca (Rebecca et al., 2007). In this study, the defatted rice bran was used and the methods was as follows (Watanabe et al., 2010): the fresh rice bran and petroleum ether were mixed at a ratio of 1:3 (m/v) and then stirred and soaked for 6 h. After that, the petroleum ether was recovered and added new one again. This process was repeated for three times. The defatted rice bran has good heat stabilization capacity and is not susceptible to oxidation and rancidity (Tang et al., 2003). Palmitic acid (PA) and oleanolic acid (OA) were purchased from Nanjing Plant Origin Biotechnology Co., Ltd. (Nanjing, China). MTT was provided by Sigma Chemical Co. (St. Louis, MO, United States). Streptozotocin (STZ) was purchased from Sigma Chemical Co. (St. Louis, MO, United States). Pioglitazone (PGLT) was obtained from Jiangsu Deyuan Pharmaceutical Co. LTD (Hunan, China). The animal high-fat diet was purchased from Trophic Animal Feed High-Tech Co., Ltd. (Jiangsu, China). Total cholesterol (TC), triglyceride (TG), low-density lipoprotein cholesterol (LDL), high-density lipoprotein cholesterol (HDL) and glucose detection kits were purchased from Jiancheng Bioengineering Institute (Nanjing, China). Superoxide dismutase (SOD), malondialdehyde (MDA), glutathione peroxidase (GSH-PX) and total antioxidant capacity (T-AOC) kits were provided by Beyotime Biotechnology Co., Ltd. (Shanghai, China). The E.Z.N.A.^®^ Stool DNA Kit was purchased from Omega Bio-tek Co. (Norcross, GA, United States). The AxyPrep DNA Gel Extraction Kit was purchased from Axygen Biosciences Co. (Union City, CA, United States).

### Preparation of the Defatted Rice Bran Fermentation Extracts

*Lactobacillus fermentum* MF423, *Lactobacillus plantarum* ZW433 and *Lactobacillus casei* GL434 were separated from Chinese rice noodle wastewater. Firstly, *Lactobacillus* fermented the defatted rice bran extracts by these three strains were screened in this study in term of the effects on IR. The results showed the *L. fermentum* MF423 fermented defatted rice bran extracts demonstrated better capacity of glucose consumption, lipid removal as well as antioxidation than extracts fermented by *L. plantarum* ZW433 and *L. casei* GL434 (Supplementary Figure 1). Thus, the *L. fermentum* MF423 was chosen to ferment the defatted rice bran.

*L. fermentum* MF423 was cultured in MRS medium. When the OD_600_ of the *L. fermentum* MF423 medium reached 0.8, the 2% (V/V) *L. fermentum* MF423 solution was inoculated into the fermentation medium (5 g of defatted rice bran, 0.1 g of Na_2_HPO_4_, 0.03 g of KH_2_PO_4_, 0.1 g of CaCl_2_, 0.1 g of Na_2_CO_3_ and 50 mL of distilled water) and cultured in static incubator at 37°C for 24 h. Finally, the supernatant was collected by centrifugation (12,000 rpm, 30 min, 4°C) and freeze-dried into powders before use. The defatted rice bran unfermented extracts (RB) were prepared as above method without *L*. *fermentum* MF423, which were considered the control group.

### Cell Culture

The human HepG2 cell line was supplied by Central South University (Changsha, China). HepG2 cells were maintained in Dulbecco’s modified Eagle’s medium (DMEM, Biological Industries, Israel), which was supplemented with 10% fetal bovine serum (FBS, v/v; MRC, Uruguay) and 1% pen-strep solution (v/v, Biological Industries, Israel) at 37°C in a 5% CO_2_ environment. HepG2 cells were harvested for the following experiments when they grew to logarithmic phase.

### Cell Viability Assay

HepG2 cells were seeded in 96-well plates (5 × 10^3^ cells per well) and cultured in an incubator for 24 h. Then, the cells were treated with FLRB (10, 100 and 200 μg/mL) and RB (10, 100, and 200 μg/mL) for 24 h. Cells treated with DMEM were used as a negative control group (NC). Then, cell viability was examined by the MTT assay (Jin et al., 2020). The absorbance was measured at 490 nm by using a multimode plate reader (Perkin Elmer, United States). The absorbance of NC cells was considered 100%.

### Insulin Resistant Model and Glucose Consumption Assay

To investigate the effect of FLRB on glucose and lipid metabolism, an insulin resistant (IR) HepG2 cell model was established according to a method with slight modifications (Xu et al., 2020). Briefly, HepG2 cells were seeded in 96-well plates (3 × 10^3^ cells per well) and cultured at 37°C for 24 h. Then, IR cells were induced with PA (0.15 μM) plus OA (0.20 μM) in serum-free and phenol red-free medium for 12 h. FLRB was dissolved in sterile water and diluted to various concentrations at 25, 50 and 100 μg/mL with DMEM and added to IR cells for 24 h. The concentration of RB was 100 μg/mL, and non-treated cells were used as a blank control. The main aim of this study is to explore the effect of the defatted rice bran fermentation extracts (FLRB) on IR. Here, the RB was used at a high dose of 100 μg/mL as the negative control group. The results of high dose (100 μg/mL) in RB group showed no function on IR, so that the lower doses (25 and 50 μg/mL) were even less effective. This comparison could also indicate that the effect of fermented products was superior to that of unfermented groups. Thus, the dose of 100 μg/mL in RB group was used in this study.

The supernatant of the culture medium was collected to measure glucose consumption. First, after the end of the experiment, the glucose level in the medium was tested according to the manufacturer’s instructions of the glucose assay kit. Then, the wells without cells were used as blank control and the glucose consumption was calculated following the formula: glucose consumption level = (glucose level in blank control group - glucose level in experiment group)/MTT value of experiment group.

### Effects of FLRB on Lipid Accumulation

After establishment of the IR HepG2 cell model, cells stained with Oil Red O were used to analyze the effect of FLRB on lipid accumulation. Briefly, FLRB (25, 50, and 100 μg/mL) and RB (100 μg/mL) were added to IR cells for 24 h, and then the medium was removed. Cells were fixed with 4% paraformaldehyde for 30 min after they were washed three times with PBS. After staining with Oil Red O solution for 10 min, the cells were washed three times with PBS. Next, 60% isopropanol was used for cell differentiation for 10 s. Finally, the cells were imaged under an inverted microscope (Zeiss, China).

### Animals and Experimental Design

Male C57BL/6J mice (6 weeks) were provided by the Animal Center of Central South University (Hunan, China). A total of 60 mice (each group consisted of 10 mice) were housed under standard conditions at a temperature of 24 ± 1°C and humidity of 55 ± 5%. Mice had free access to water and food. The animal experiment procedures in this study were conducted in accordance with the guidelines of the Care and Use of Laboratory Animals and were approved by the Ethics Committee of School of Life Sciences, Central South University (Changsha, China).

The T2DM mouse model was developed following a previously published approach (Zhang B. W. et al., 2018). In the first 8 weeks of this experiment, mice in the negative group (NC) were fed normal chow, and other groups were fed a high-fat diet. At the eighth week, mice fed a high-fat diet were intraperitoneally injected with STZ (35 mg/kg body weight) to compromise the function of islet β-cells to induce insulin resistance. At the same time, mice in the NC group were injected with citrate buffer solution (0.1 mL/10 g body weight). Then, fasting blood glucose was measured on days 3 and 7 after intraperitoneal injection. The mice were defined as T2DM if the fasting blood glucose was more than 7.0 mmol/L (Wang J. et al., 2020). And T2DM mice were randomized into five groups: high-fat diet (HFD, 0.1 mL/10 g body weight, distilled water), defatted rice bran unfermented extracts (RB, 1.0 g/kg body weight), pioglitazone (PGLT, 10 mg/kg body weight, positive control), high-dose of defatted rice bran fermentation extracts (HFLRB, 1.0 g/kg body weight), and low-dose of defatted rice bran fermentation extracts (LFLRB, 0.5 g/kg body weight). All mice were treated through intragastric administration. Body weight and fasting blood glucose (FBG) were measured weekly during the experiment. The experiment scheme is shown in Supplementary Figure 2.

### Oral Glucose Tolerance Test

All mice were orally administered 2 g/kg glucose individually after a 12-h fast. Blood glucose was tested using a Roche glucometer via collection from the tail vein at a series of time points of 0, 30, 60, and 120 min after glucose intake.

### Biochemical Analysis

At the end of the study, all mice were sacrificed with isoflurane after a 12-h fast. The serum was obtained by centrifuging at 4,000 rpm for 10 min at 4°C. The liver tissues were rinsed with saline to produce liver homogenate with anhydrous ethanol. The levels of TC, TG, LDL, HDL, SOD, MDA, GSH-PX and T-AOC were quantified using corresponding detection kits according to the manufacturer’s protocols. For histological study, the liver segments were immediately fixed with 4% polyoxymethylene and stained with hematoxylin and eosin (H&E) (Servicebio, China).

### Fecal Sample Collection and DNA Extraction

Individual sterile Eppendorf tubes were used to collect fresh mouse feces, which were then immediately frozen on ice and stored at −80°C for further measurement. Microbial DNA was extracted from 24 fecal samples (NC, HFD, PGLT and HFLRB groups, with 6 mice in each group) by using the E.Z.N.A.^®^ Stool DNA Kit following the manufacturer’s protocols. The concentration and purity of DNA samples were determined with a NanoDrop 2000 UV-vis spectrophotometer (Thermo Scientific, Wilmington, United States).

### 16S rRNA Gene Fragment Sequencing

Hypervariable V3 and V4 regions of the prokaryotic 16S rRNA gene in DNA samples were selected for amplification with universal primers (338F 5’-ACTCCTACGGGAGGCAGCAG-3’, 806R 5’-GGACTACHVGGGTWTCTAAT-3’) by an ABI GeneAmp^®^ 9700 PCR thermocycler (ABI, CA, United States). The PCR amplification procedure was as follows: 95°C for 3 min; 27 cycles of 30 s at 95°C for denaturing, annealing at 55°C for 30 s, and extension at 72°C for 45 s; and a single extension at 72°C for 10 min. The AxyPrep DNA Gel Extraction Kit (Axygen Biosciences, United States) and QuantiFluor^TM^-ST (Promega, United States) were used to purify and quantify the PCR products, respectively, which were extracted from a 2% agarose gel. The purified amplicons were pooled in equimolar amounts and paired-end sequenced on an Illumina MiSeq PE300 platform/NovaSeq PE250 platform (Illumina, San Diego, United States) according to the standard protocols by Majorbio Bio-Pharm Technology Co. Ltd. (Shanghai, China).

### Bioinformatics Analysis

The trimmed data were clustered into operational taxonomic units (OTUs) at 97% similarity by using UPARSE (Edgar, 2013), and chimeric sequences were confirmed and removed using UCHIME (Robert et al., 2011). The taxonomic assignment of the 16S rRNA gene sequence was conducted through the RDP classifier algorithm^1^ against the Silva database (Quast et al., 2013) using a confidence threshold of 70%.

The Mothur program (Schloss et al., 2009) was used to analyze alpha-diversity, including richness estimators (ACE and Chao) and diversity indices (Shannon and Simpson). Variations in microbiota communities were evaluated using similarities analysis (AMOSIM), principal coordinate analysis (PCoA) and NMDS analysis (Majorbio Bio-Pharm Technology Co. Ltd. Shanghai, China)^2^. The distributions of microbiota community compositions at different taxonomic levels were generated by using a hierarchical clustering tree and Venn diagram. The linear discriminant analysis (LDA) effect size (LEfSe) method was used to compare the microbiota diversity among 24 fecal samples. This approach was calculated based on non-parametric testing of independent samples (Kruskal-Wallis H test). The raw 16S rRNA gene sequencing reads were demultiplexed and quality-filtered by fastp version 0.20.0 (Chen S. et al., 2018) and merged by FLASH version 1.2.7 (Magoč and Salzberg, 2011) with the following criteria: (i) the 300 bp reads were truncated at any site receiving an average quality score of <20 over a 50 bp sliding window, and the truncated reads shorter than 50 bp were discarded, reads containing ambiguous characters were also discarded; (ii) only overlapping sequences longer than 10 bp were assembled according to their overlapped sequence. The maximum mismatch ratio of overlap region is 0.2. Reads that could not be assembled were discarded; (iii) Samples were distinguished according to the barcode and primers, and the sequence direction was adjusted, exact barcode matching, 2 nucleotide mismatches in primer matching. The false discovery rates were used with the statistical analysis alongside identification of *P*-values of *P* < 0.05. The relationship between the microbiota diversities and biochemical parameters was assessed by using Spearman correlation analysis.

### Fecal SCFA Measurement by GC-MS

The fecal samples (200 mg) were ultrasound extracted with diethyl ether (450 μL) and centrifuged at 12,000 rpm at 4°C for 10 min. The supernatant was moved to a new centrifuge tube for natural drying, and methyl alcohol was added to dissolve. Next, the samples were filtered through a 0.22-μm membrane. The filtrate was injected into a GC-MS apparatus (Agilent, United States) for SCFA measurement.

### Statistical Analysis

Statistical analysis was carried out with R software, and all data are presented as the mean ± standard difference (SD). Statistics between two groups were conducted by using unpaired Student’s t test, and one-way ANOVA followed by a Tukey test was used to analyze more than two groups. All data analyses were two-tailed, and a *P*-value <0.05 was considered statistically significant.

## Results and Discussion

### Effects of FLRB on Glucose Consumption in IR-HepG2 Cells

**FIGURE 1:**
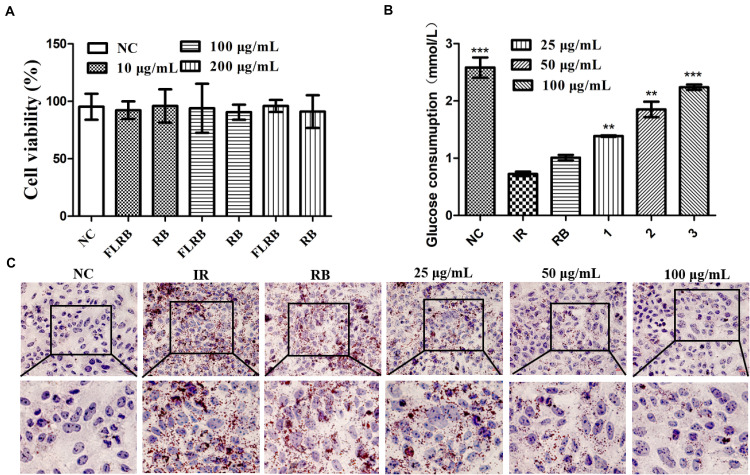
Analysis of the effects of FLRB *in vitro*. Cells were treated with FLRB and RB at various concentrations. **(A)** HepG2 cell viability was examined by the 3-(4,5-dimethylthiazol-2-yl)-2,5-diphenyltetrazolium bromide (MTT) assay; **(B,C)** glucose consumption and lipid accumulation assay of *Lactobacillus fermentum* MF423-fermented rice bran extracts (FLRB) at 25, 50, and 100 μg/mL was determined in insulin resistant-HepG2 (IR-HepG2) cells for 24 h; the concentration of unfermented rice bran extracts (RB) used was 100 μg/mL. Values are expressed as the mean ± SD (*n* = 3). *** represents *P* < 0.001, ** represents *P* < 0.01, compared to the IR.

*L. fermentum* MF423 was used to ferment the defatted rice bran in this study. To explore the effects of FLRB on glucose consumption, we used HepG2 cells to establish a cell insulin resistance model for analysis. First, cell viability was examined by the MTT assay to determine the cytotoxic effects of FLRB on HepG2 cells. The results indicated that there were no significant toxic effects of FLRB at 10–200 μg/mL on HepG2 cell viability after 24 h (Figure 1A). Therefore, further experiments were performed with FLRB at 25–100 μg/mL.

A high level of glucose is usually observed in an insulin-resistant model (Solis et al., 2021). To some extent, increased glucose consumption can improve IR. Glucose consumption was detected in HepG2 cells treated with PA and OA to verify whether FLRB could improve insulin resistance. The IR-HepG2 cell model was developed following the above method (Xu et al., 2020). As shown in Figure 1B, the glucose consumption of cells treated with DMEM containing PA (0.15 μM) and OA (0.2 μM) was significantly lower than that of cells treated with normal DMEM, indicating that coincubation with PA and OA could induce insulin resistance in HepG2 cells. Nevertheless, the glucose consumption of cells treated with FLRB was markedly higher than that of the PBS-treated negative control group cells and showed a dose-dependent pattern (*P* < 0.01 or *P* < 0.001) (Figure 1B). There was no difference in glucose consumption between RB-treated cells and IR-HepG2 cells (Figure 1B). These results suggested that the defatted rice bran fermentation broths could enhance glucose consumption in IR-HepG2 cells. Herein, we may provide a feasible approach for improving glucose consumption in IR by fermenting the defatted rice bran.

### Effects of FLRB on Lipid Accumulation in IR-HepG2 Cells

In T2DM, hyperlipidemia is closely linked to insulin resistance, which involves a complex network of signaling pathways activated by insulin receptor-regulated intermediary metabolism (Saltiel and Kahn, 2001). Oil Red O staining of FLRB-treated IR-HepG2 cells was used to determine the effects on lipid accumulation. As shown in Figure 1C, compared with the control group, many more lipid droplets accumulated in IR-HepG2 cells, suggesting that the IR cell model was successfully established. FLRB treatments caused a notable decrease in lipid droplets in a dose-dependent manner, while no effect was observed in RB-treated cells (Figure 1C). Our findings confirmed that FLRB could significantly improve lipid accumulation in IR-HepG2 cells. Thus, we speculate that there may be certain components in rice bran fermentation broth that could ameliorate lipid metabolism to improve insulin resistance.

### The Effect of FLRB Supplementation on Body Weight in Mice

**FIGURE 2:**
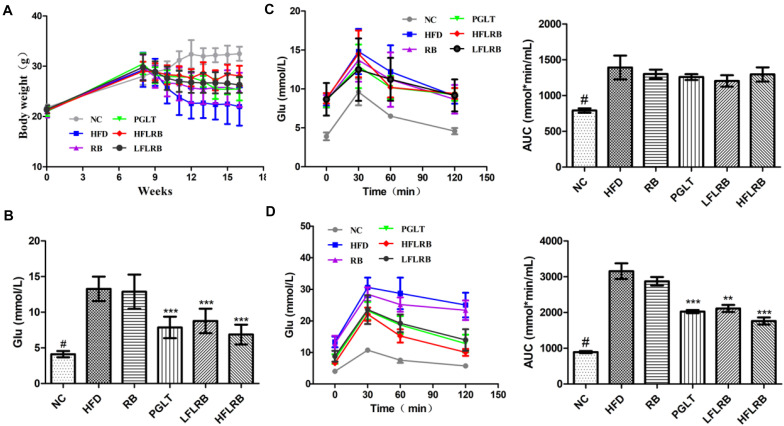
Effects of FLRB on body weight and insulin resistance. Diabetic mice were treated with FLRB (1.0 and 0.5 g/kg), pioglitazone (PGLT) (10 mg/kg) and RB (1.0 g/kg) for 8 weeks. **(A)** Effect of body weight; **(B)** effect of blood glucose; **(C,D)** results of the Oral glucose tolerance test (OGTT) at the 8th and 16th weeks. Values are expressed as the mean ± SD (*n* = 10). ^#^ and *** represent *P* < 0.001, ** represents *P* < 0.01, compared to the high-fat diet (HFD).

As mentioned above, we demonstrated that FLRB could increase glucose consumption and decrease lipid accumulation *in vitro*, indicating that defatted rice bran would gain anti-insulin resistance activity after it was fermented by *Lactobacillus fermentum* MF423 (Figure 1). Therefore, to further evaluate the potential of FLRB on IR, mouse T2DM syndrome was successfully established according to the literature (Zhang B. W. et al., 2018). In this study, we measured the body weight of mice weekly. During the first 8 weeks, body weight was persistently increased in all mice (Figure 2A). However, a significant weight loss was observed in all high-fat diet groups from 8 weeks to 10 weeks after the injection of STZ, while FLRB and PGLT distinctly slowed the rate of weight loss, with significant differences compared to the HFD group (Figure 2A). These results indicated that FLRB supplementation could inhibit the degree of weight loss.

### Effect of FLRB on Glucose Tolerance in Mice

After the injection of STZ and high-fat diet induction, the levels of fasting blood glucose (FBG) were obviously higher in the HFD mice than in the normal mice (*P* < 0.001) (Figure 2B). After an 8-week treatment, the FBG in the FLRB group and PGLT group was clearly reduced compared to that in the HFD group, which was similar to that of the NC group (*P* < 0.001) (Figure 2B). However, the blood glucose levels of mice in the RB and HFD groups were extraordinarily similar (Figure 2B). The oral glucose tolerance test (OGTT) is a recognized criterion for insulin resistance in T2DM (Kodaganti et al., 2017). In this study, an OGTT was used to determine the effects of defatted rice bran products on glucose tolerance in T2DM mice. At the 8th week, glucose tolerance was dramatically damaged in high-fat diet-fed mice, and the area under the curve (AUC) value was significantly higher than that in the NC group (*P* < 0.001) (Figure 2C). After the 8-week treatment, FLRB (1.0 and 0.5 g/kg) and PGLT mice showed an obvious improvement in glucose tolerance, along with a decrease in blood glucose levels (*P* < 0.01 or *P* < 0.001) (Figure 2D). These results demonstrated that FLRB, especially at a dose of 1.0 g/kg, has the potential to reinforce glucose tolerance in T2DM mice. A previous study also showed that lactobacilli-fermented products could lower blood glucose levels and improve glucose tolerance in STZ-induced diabetic rats (Kim et al., 2010), which was consistent with our study. Here, fermentation by *Lactobacilli* may be a good method to increase the bioactivity of defatted rice bran, and its fermentation broth may be considered a potential probiotic for T2DM.

### Improvement in the Lipid Profile by FLRB in Mice

**FIGURE 3:**
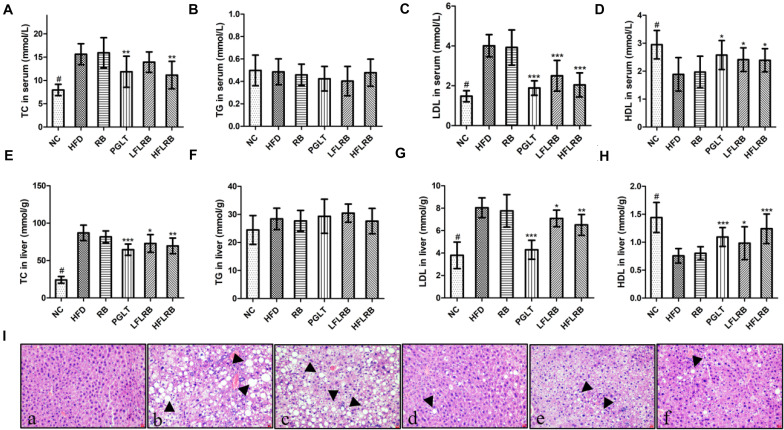
Effects of FLRB on the lipid profile in mice. **(A–H)** Total cholesterol (TC), triglyceride (TG), low-density lipoprotein cholesterol (LDL), high-density lipoprotein cholesterol (HDL) levels in serum and liver; **(I)** hematoxylin and eosin (H&E) staining of liver tissue. **(a)**: negative group (NC), **(b)**: HFD, **(c)**: RB, **(d)**: PGLT, **(e)**: LFLRB, **(f)**: HFLRB. Representative results are shown. Scale bar: 2 μm. Values are expressed as the mean ± SD (*n* = 10). ^#^ and *** represent *P* < 0.001, ** represents *P* < 0.01, * represents *P* < 0.05, compared to the HFD.

At the end of the research, the lipid levels were measured, and the results are described in Figures 3A–H. Compared with the control mice, the TC and LDL levels were significantly enhanced in the high-fat diet mice (*P* < 0.001) (Figures 3A,C,E,G), and the HDL level was obviously decreased in the HFD group in both the serum and liver (*P* < 0.001) (Figures 3D,H). However, administration of FLRB significantly decreased TC and LDL levels and increased HDL levels in both the serum and liver of T2DM mice (*P* < 0.05, *P* < 0.01, or *P* < 0.001) (Figures 3A,C,E,G). RB had no effect on lipid metabolism in either the serum or liver (Figures 3A,C,E,G). There was no change in the levels of TG in any group (Figures 3B,F). These results indicated that FLRB could improve lipid levels in a dose-dependent manner. Previous studies have shown that lactic acid bacteria fermentation products can decrease blood lipid levels by modulating related genes involved in lipid metabolic signaling pathways (Zhang et al., 2019; Chen et al., 2020). In our study, the active ingredients of fermented defatted rice bran may also be involved in the regulation of lipid metabolism pathway genes to reduce blood lipids and glucose, and the exact mechanism needs further study.

### Liver Histological Analysis

The liver is a crucial organ for glucose and lipid metabolism. A previous study showed that hyperglycemia is closely associated with liver damage and hepatic steatosis (Chen et al., 2017). As shown in Figure 3I, abnormal hepatocytes and hepatic steatosis (black arrows) were significantly increased in the HFD and RB groups, whereas the NC group had normal cell structure and morphology, suggesting that hepatocytes may have been damaged by the high-fat diet. In contrast, FLRB and PGLT treatment ameliorated the damage to liver cells and significantly reduced the accumulation of lipid droplets, similar to the NC group in diabetic mice (Figure 3I). The H&E staining results implied that a good liver protection effect might be revealed with the FLRB treatment in T2DM mice.

### FLRB Intensifies Antioxidant Activity in Mice

**FIGURE 4:**
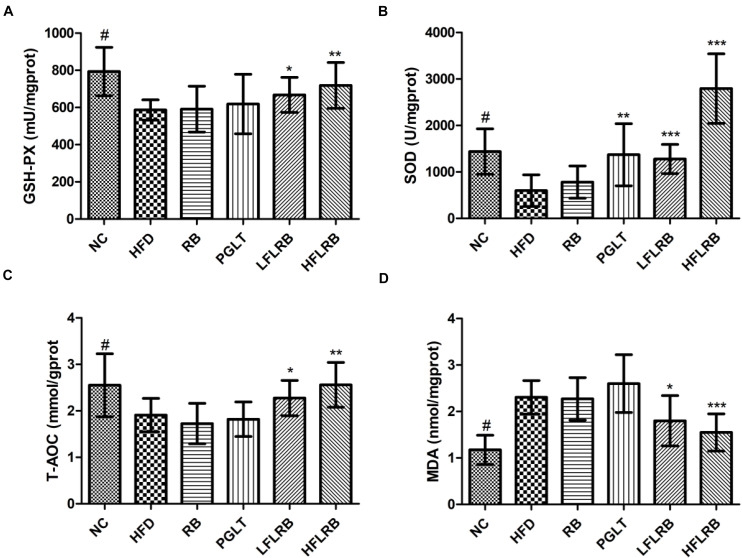
Effects of FLRB on the antioxidant activity in the livers of mice. **(A)** Glutathione peroxidase (GSH-PX), **(B)** Superoxide dismutase (SOD), **(C)** total antioxidant capacity (T-AOC), **(D)** malondialdehyde (MDA). Values are expressed as the mean ± SD (*n* = 10). ^#^ and *** represent *P* < 0.001, ** represents *P* < 0.01, * represents *P* < 0.05, compared to the HFD.

The development of insulin resistance affects oxidative stress in diabetic mice, leading to a significant increase in the levels of reactive oxygen species (ROS) and MDA in mice (Li et al., 2021). Previous studies have proven that a large number of antioxidant enzymes in organisms, such as SOD, T-AOC and GSH-PX, can suppress oxidative stress by scavenging ROS levels (Liu S. W. et al., 2020; Wu et al., 2021). The liver is the largest metabolic organ possessing abundant antioxidant enzymes. Therefore, the levels of several antioxidant enzymes in the liver were determined. Our results showed that the levels of SOD, T-AOC and GSH-PX were significantly reduced in the HFD group compared with the NC group, but MDA levels were obviously higher (*P* < 0.001) (Figure 4). However, the administration of FLRB significantly upregulated the levels of SOD, T-AOC and GSH-PX compared with the HFD group (*P* < 0.05, *P* < 0.01, or *P* < 0.001) (Figures 4A–C). Moreover, the elevation of MDA was significantly reversed by treatment with FLRB in diabetic mice (*P* < 0.05 or *P* < 0.001) (Figure 4D). Compared with the HFD group, no such effects were found in the RB group (Figure 4). These results suggested that administration of FLRB in T2DM mice activates the antioxidant system in their bodies, such as upregulating SOD, which may be one of the reasons for the protection of the liver in the FLRB group of mice.

### Alpha- and Beta-Diversity of Microbiota Communities in Fecal Samples

Based on the above results, we found that FLRB could vastly improve hyperglycemia and hyperlipidemia both *in vitro* and *in vivo* and that it exerted high-efficiency anti-insulin resistance activity. To further explore the possible mechanisms involved, we explored the modulation of gut microbiota by FLRB in diabetic mice. The raw sequencing data were obtained by next-generation sequencing with 16S rRNA gene amplicons on fecal samples collected from 24 mice (NC = 6, HFD = 6, PGLT = 6 and HFLRB = 6). A total of 1,092,405 valid sequences were obtained after quality control (Supplementary Table 1). The rarefaction curves showed that the sequencing data depth was comprehensive, covering most of the microorganisms in all samples (Supplementary Figure 3A). The sequencing coverage of all samples was 0.99 (Supplementary Figure 3B), which could be used for subsequent data analysis. In addition, 360 OTUs were matched in all samples, including 10 phyla, 118 genera and 183 species of gut microbiota that were subjected to subsequent analysis.

To assess the alterations in the intestinal microbiota community structure among the four groups, alpha-diversity was evaluated by using the Shannon, Ace, Simpson and Chao diversity indices. Although there were no significant differences between the groups, the Shannon, Ace and Chao diversity indices were higher in the NC group and HFLRB group than in the HFD group and PGLT group, and the opposite trend was observed for the Simpson index (Supplementary Figures 3C–F). The above results showed that the gut microbiota was more diverse in healthy mice than in diabetic mice. The administration of FLRB (1.0 g/kg) may increase diversity of the gut microbiota in diabetic mice.

In contrast, beta-diversity analysis was used to evaluate the variations in the intestinal microbiota in the four groups and showed significant differences (Supplementary Figures 4A–C). As shown in Supplementary Figure 5, the similarities between the groups were assessed by AMOSIM analysis, indicating that the intergroup differences were more significant than the intrasample differences (*P* < 0.01). In addition, principal coordinate analysis (PCoA) and non-metric multidimensional scaling analysis (NMDS) based on Bray-Curtis arithmetic at the OTU level were used to demonstrate the overall structural alteration of the intestinal microbiota, which presented distinct separation between normal mice and other diabetic mice (R = 0.8796, *P* = 0.001), and the selected coordinate components (PC1 and PC2) were 24.14 and 19.75% of the total variations, respectively (Supplementary Figures 4A,B). The gut microbiota in the HFD group exhibited a significant change compared to the NC group, while the HFLRB and PGLT groups showed much alteration in the opposite direction, which was more similar to that of the NC group (Supplementary Figures 4A,B). In addition, cluster analysis also indicated these trends in the four groups (Supplementary Figure 4C). Compared with the HFD group, the FLRB treatment group showed a significant change in gut microbiota, and the composition was closer to that of the NC and PGLT groups (Supplementary Figure 4C). These results suggested that FLRB treatment could significantly reverse the changes in gut microbiota compositions in the HFD group.

### Taxonomic Compositions and Relative Abundance of Microbiota Communities in Different Fecal Samples

**FIGURE 5:**
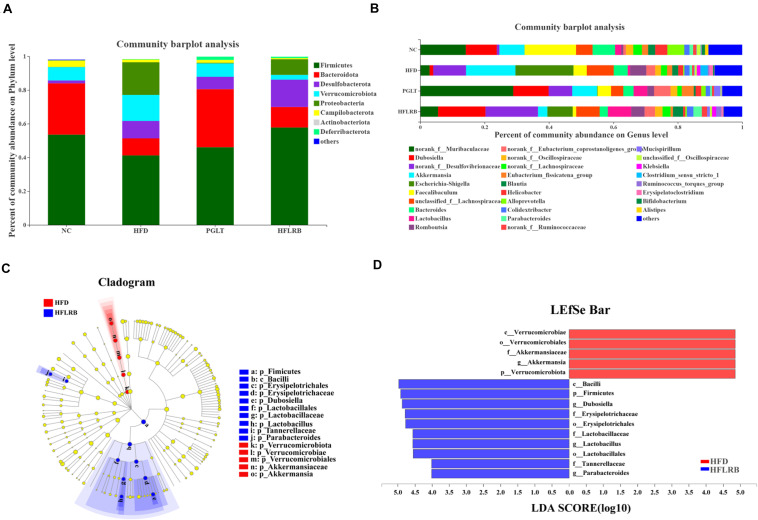
The distributions of microbial community compositions in diabetic mice at the **(A)** phylum level and **(B)** genus level. The distributions at the genus level are shown as the average relative abundances of the top 30 genera in each sample. **(C)** The circle midpoint represents indicator bacteria with LDA scores greater than 4.0 in microbial groups. Yellow dots represent microbes with no significant differences among the 4 groups. **(D)** Histogram of LDA scores computed for differentially abundant microbes among the 4 groups.

To further investigate which microbial taxa are affected by the fermentation products, taxonomic compositions and relative abundances of microbiota communities at different taxonomic levels were examined. At the phylum level, the microbial community structure was broadly similar in the four groups of mice, including Firmicutes, Bacteroidota, Desulfobacterota, Verrucomicrobiota, Proteobacteria, Campilobacterota, Actinobacteriota and Deferribacterota (Figure 5A). Our data showed that the gut microbiota in mice was largely dominated by the phyla Firmicutes and Bacteroidota (Supplementary Table 2). Microflora in diabetic mice were sharply shifted, with a reduction in the proportion of the phylum Bacteroidota by 66% and the phylum Firmicutes by 23%. Compared to the HFD group, the relative abundances of Bacteroidetes (20%) and Firmicutes (40%) were increased in the HFLRB group, similar to the results of the PGLT group (Figure 5A). Moreover, the taxonomic groups Verrucomicrobiota and Proteobacteria were obviously lower in HFLRB-treated mice than in HFD mice. Several studies found that the abundance of Firmicutes and Bacteroidota was decreased in T2DM mice (Anhe et al., 2015; Chen et al., 2019), indicating that these two major phyla may play an essential role in hyperglycemia, hyperlipidemia and inflammation level (Cani et al., 2008, 2009). Similarly, compared to the healthy controls, the reduction of Firmicutes and Bacteroidota was observed in T2DM patients (Ebrahimzadeh et al., 2021). In our study, the reduced abundance of Firmicutes and Bacteroidota was observed in T2DM mice, which was consistent with previous studies. However, the treatment of PGLT and FLRB significantly increased the abundance of Firmicutes and Bacteroidota (Figure 5A). These results indicated that the FLRB is promising to exert the anti-diabetic effects by regulating the structure of gut microbiota.

At the genus level, the gut microbiota composition is shown with a bar plot among the four groups (Figure 5B). Our data showed that 26 out of 30 genera were identified and changed significantly following FLRB treatment, including 14 enriched genera and 12 decreased genera. Compared to the HFD group, a variety of putative SCFA-producing bacteria were enriched in FLRB-treated mice, including *Lactobacillus, Parabacteroides, norank_f__Ruminococcaceae, Ruminococcus_torques_group* and *Alloprevotella* (Tanca et al., 2017; Zhao et al., 2017). The relative abundances of the genera *Dubosiella, norank_f__Desulfovibrionaceae, norank_f__Muribaculaceae, Blautia* and *norank_f__Lachnospiraceae* were also increased in the HFLRB group versus the HFD group (Figure 5B and Supplementary Table 3). In addition, *Akkermansia*, *Escherichia-Shigella, Faecalibaculum, Bacteroides, Clostridium_sensu_stricto_1* and *unclassified_f__Lachnospiraceae* in the HFLRB group were decreased compared with those in the HFD group (Figure 5B and Supplementary Table 3). Moreover, the LEfSe method with an LDA threshold of 4.0 was utilized to analyze the predominant bacteria in the HFD group and HFLRB group and is displayed as a cladogram in Figures 5C,D. We discovered the key variables that separated the intestinal microbiota under FLRB treatment and identified some phylotypes as high-dimensional biomarkers, such as *Dubosiella* and *Lactobacillus*, which was consistent with the above results.

Interestingly, the genus *Lactobacillus* (Lactobacillales order, Lactobacillaceae family) was significantly decreased in diabetic mice, while treatment with FLRB increased it by approximately eightfold, which was much higher than it was in NC mice (Figure 5B and Supplementary Table 3). *Lactobacillus* is known for its probiotic roles in food consumption, which could modify abnormalities in intestinal microbes and retard hyperglycemia (Liu Z. G. et al., 2020). Moreover, *Lactobacillus* was also demonstrated to be obviously associated with butyrate levels (Sun et al., 2020). Our results showed that FLRB further promoted the growth and reproduction of *Lactobacillus* in the intestine, thus improving the composition of the gut microbiota and increasing the relative abundance of SCFA-producing bacteria. Furthermore, *Dubosiella*, belonging to the family Erysipelotrichaceae, was increased 13-fold compared to HFD mice (Figure 5B and Supplementary Table 3), suggesting that it is closely associated with remodeling of the gut microbiota. To our knowledge, *Dubosiella* was found to be significantly reduced in high-fat diet- and STZ-induced diabetic mice for the first time. Meanwhile, the feeding results suggested that treatment with FLRB restored *Dubosiella*, which may play a novel role in regulating the gut microbiota in a T2DM mouse model. To the best of our knowledge, our study was the first direct evidence to show that the relative abundance of *Dubosiella* was highly enriched in FLRB-treated diabetic mice. A previous study indicated that vitamin K2 supplementation could increase the abundance of intestinal *Dubosiella* and decrease the relative expression of activating transcription factor 4 to inhibit blood pressure in mice (Liu T. H. et al., 2020). However, changes in *Dubosiella* in the intestine of diabetic mice have not yet been reported. Our observation of the enriched *Dubosiella* in FLRB-treated mice may contribute to the improvement in insulin resistance in this study. Moreover, Spearman correlation analysis in FLRB-treated mice further confirmed its role by showing a positive correlation with HDL, GSH-PX, SOD and T-AOC levels and a negative correlation with TC, LDL and Glu levels. Therefore, we hypothesized that *Lactobacillus* and *Dubosiella* were closely associated with intestinal microbial modulation and the antidiabetic effects of FLRB in this study.

SCFAs are the main microbial fermentation products of undigested carbohydrates in the intestine (Serrano et al., 2021). Our data showed that SCFA-producing bacteria were enriched in FLRB-treated mice compared to HFD-fed mice (Figure 5B). Thus, we further investigated alterations in the content of SCFAs among the four groups. As shown in Supplementary Figure 6, the levels of butyric acid, valeric acid and total SCFAs were significantly decreased in the HFD group compared with the NC group. In contrast, FLRB obviously retarded the reduction in SCFAs in diabetic mice, but it was still lower than that in the NC group (*P* < 0.05 or *P* < 0.01) (Supplementary Figure 6). However, there were no significant changes in valeric acid or total SCFAs in the PGLT group compared with the HFD group (Supplementary Figures 6A,C). These results suggested that the administration of FLRB could increase the content of SCFAs in diabetic mice and might account for the change in gut microbiota in this study. SCFAs were proven to play a crucial role in gut barrier function by improving epithelial growth and can impede appetite by upregulating several proteins related to satiety, and they were closely correlated with the structure of intestinal microbes (Kim et al., 2018; Liu et al., 2021). In our study, we showed that the treatment of defatted rice bran fermentation extracts not only enriched SCFA-producing bacteria but also increased the content of SCFAs in diabetic mice, suggesting that the major modulation of intestinal microbes may occur by changing the production of SCFAs. Hence, FLRB may be considered a valuable dietary supplement for T2DM.

### Relationship Between Microbiota Communities and Biochemical Parameters

**FIGURE 6:**
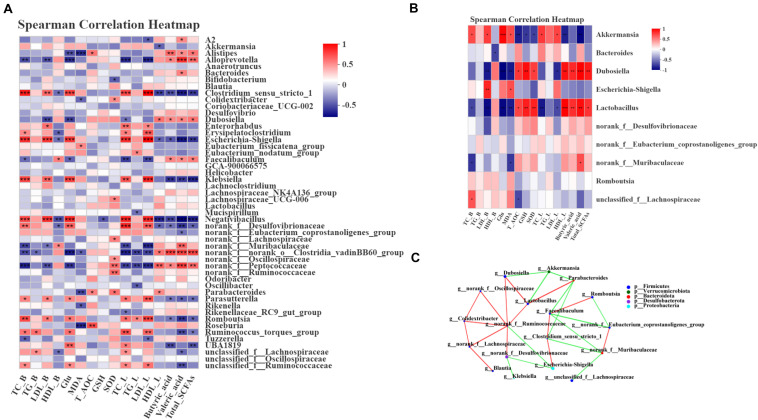
Spearman correlation between key phylotypes, their relative abundance, and biochemical parameters. **(A)** Correlation between phylotypes and biochemical parameters of samples from the NC, HFD, PGLT and HFLRB groups; **(B)** correlation between the HFD and HFLRB groups. *P* values for the correlations are calculated: * represents *P* < 0.05, ** represents *P* < 0.01, *** represents *P* < 0.001. A positive correlation is labeled in red, and a negative correlation is labeled in blue. **(C)** Cooccurrence network analysis of the 18 most abundant genera deduced from two groups of diabetic and FLRB-treated diabetic mice. The size of the nodes indicates the number of phylotypes. The lines connecting each node represent the Spearman correlation coefficient values that were above 0.4 (red) or below –0.4 (green).

Spearman correlation analysis was used to assess whether there was a potential relationship between the host phenotypes and the alteration of intestinal microbiota. First, we analyzed the relative abundance of the 50 most abundant genera in the 4 groups. *Dubosiella*, *norank_f__Muribaculaceae* and *Alloprevotella* showed significantly positive correlations with HDL and SCFA contents and negative correlations with TC, LDL and Glu (*P* < 0.05, *P* < 0.01, or *P* < 0.001) (Figure 6A). *Parabacteroides, norank_f__Ruminococcaceae* and *norank_f__Lachnospiraceae* showed significant positive correlations with GSH, T-AOC and SOD levels (*P* < 0.05, *P* < 0.01, or *P* < 0.001) (Figure 6A). Moreover, *Escherichia-Shigella* and *Clostridium_sensu_stricto_1* were notably positively correlated with TC, LDL and Glu and negatively correlated with the SCFA contents and HDL (*P* < 0.05, *P* < 0.01, or *P* < 0.001) (Figure 6A). Correlation analysis between the treatment group and the model group was an efficient approach to uncover the potential impact of drug-modulated phylotypes on host phenotypes. Thus, we further analyzed the correlation using 12 samples from the HFLRB group and HFD group. The data in *Dubosiella* and *Lactobacillus* showed a significant positive correlation with the SCFA contents, HDL, GSH-PX, SOD and T-AOC and a negative correlation with TC, LDL and MDA (*P* < 0.05, *P* < 0.01, or *P* < 0.001) (Figure 6B). *Akkermansia* showed a notable positive correlation with TC, LDL, MDA and Glu and a negative correlation with the SCFA contents, GSH-PX, T-AOC, SOD and HDL (*P* < 0.05, *P* < 0.01, or *P* < 0.001) (Figure 6B). Based on the above results, we presume that the gut microbiota remodeled by FLRB treatment in diabetic mice might play a key role in glucose and lipid metabolism and modulate the effects of antioxidants.

To investigate whether there is a potential relationship between the relative abundance of different key phylotypes on host phylotypes, the Spearman correlation method was used for analysis. As shown in Figure 6C, all the samples from the HFD and HFLRB groups were analyzed, and the results indicated the correlations among the 18 most abundant genera. *Norank_f__Ruminococcaceae* showed a positive correlation with *Lactobacillus, Dubosiella, norank_f__Desulfovibrionaceae, norank_f__Oscillospiraceae, Colidextribacter* and *norank_f__Lachnospiraceae* and a negative correlation with *Escherichia-Shigella* (Figure 6C). *Lactobacillus* showed a positive correlation with *Dubosiella* and *Parabacteroides* and a negative correlation with *Akkemansia* (Figure 6C). *Norank_f_Muribaculaceae* showed a positive correlation with *norank_f_Eubacterium_coprostanoligenes_group* and a negative correlation with *unclassified_f_Lachnospiraceae* and *Clostridium_sensu_stricto_1.* These results indicated that the different key phylotypes showed close correlations with each other, which might play a certain role in regulating the intestinal microbiota.

## Conclusion

Rice bran contains a mass of protein, dietary fiber, phenols and γ-oryzanol, which have been demonstrated to have antidiabetic, anti-dyslipidemia and antioxidant properties both *in vivo* and *in vitro*. However, rice bran has not yet been regarded as a food supplement for human consumption because it is limited in use, resulting in substantial waste. Our study showed that FLRB extracted from defatted rice bran fermentation by *Lactobacillus fermentum* MF423 could improve hyperglycemia and hyperlipidemia both *in vitro* and *in vivo* and enhance the antioxidant capacity of diabetic mice. Moreover, FLRB could reshape the structure of the gut microbiota in diabetic mice to one more closely resembling that in normal littermates, increasing the contents of SCFAs in feces and enriching some SCFA-producing bacteria. Based on the above results, our study may offer novel insights into the potent glucose-lowering and lipid-lowering activities of FLRB from a gut microbiota perspective. These effects might be beneficial to further extend the application of rice bran as a potential prebiotic or dietary supplement for T2DM treatment.

## Data Availability Statement

The raw sequence data reported in this paper have been deposited in the Genome Sequence Archive (Genomics, Proteomics & Bioinformatics 2017) in National Genomics Data Center (Nucleic Acids Res 2021), China National Center for Bioinformation/Beijing Institute of Genomics, Chinese Academy of Sciences, under accession number CRA003897 that are publicly accessible at https://bigd.big.ac.cn/gsa.

## Ethics Statement

The animal study was reviewed and approved by the Ethics Committee of School of Life Sciences, Central South University.

## Author Contributions

XA performed the experiments and prepared the manuscript draft. HH, CY, and CW conceived and designed the experiments. HH, BX, and XY helped revise the manuscript. DL, LX, and OZ assisted in formatting figures. CL, WL, and TY assisted in data analysis. All authors read and agreed to the final content.

## Conflict of Interest

The authors declare that the research was conducted in the absence of any commercial or financial relationships that could be construed as a potential conflict of interest.

## Footnotes

1 http://rdp.cme.msu.edu/

2 www.i-sanger.com
