# Supplementary material for: Antidiabetic Function of Lactobacillus fermentum MF423-Fermented Rice Bran and Its Effect on Gut Microbiota Structure in Type 2 Diabetic Mice
Source: Front Microbiol. 2021 Jun 24;12:682290. doi: 10.3389/fmicb.2021.682290 (PMC8266379; doi:10.3389/fmicb.2021.682290)
Supplement: Supplementary file 1 [file Table_1.DOCX]

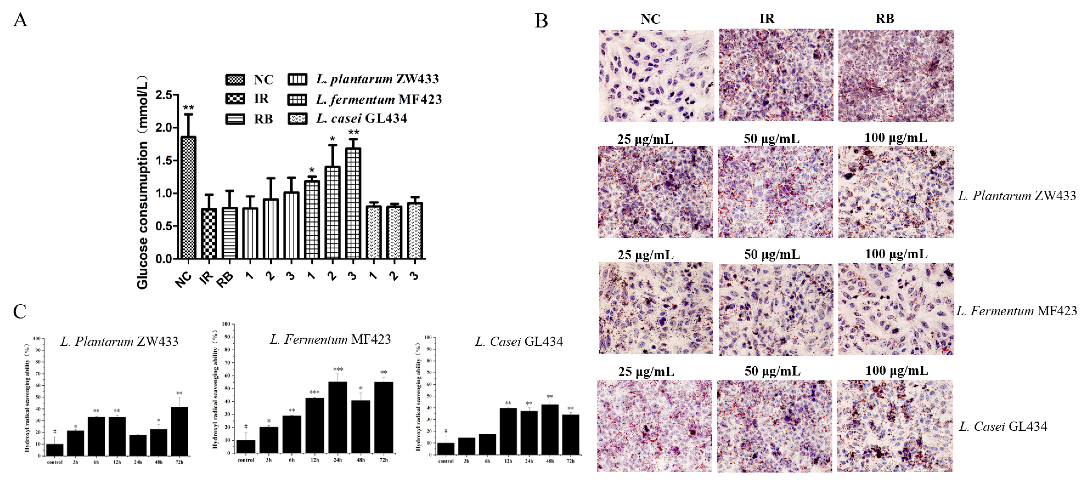


**Figure S1. Strain screening assay.** (A) Glucose consumption assay, 1, 2, 3 represent 25, 50 and 100 μg/mL, respectively. (B) Lipid accumulation assay. (C) Hydroxyl free radical assay. NC: negative control; IR: insulin resistance model; RB: unfermented rice bran extracts.


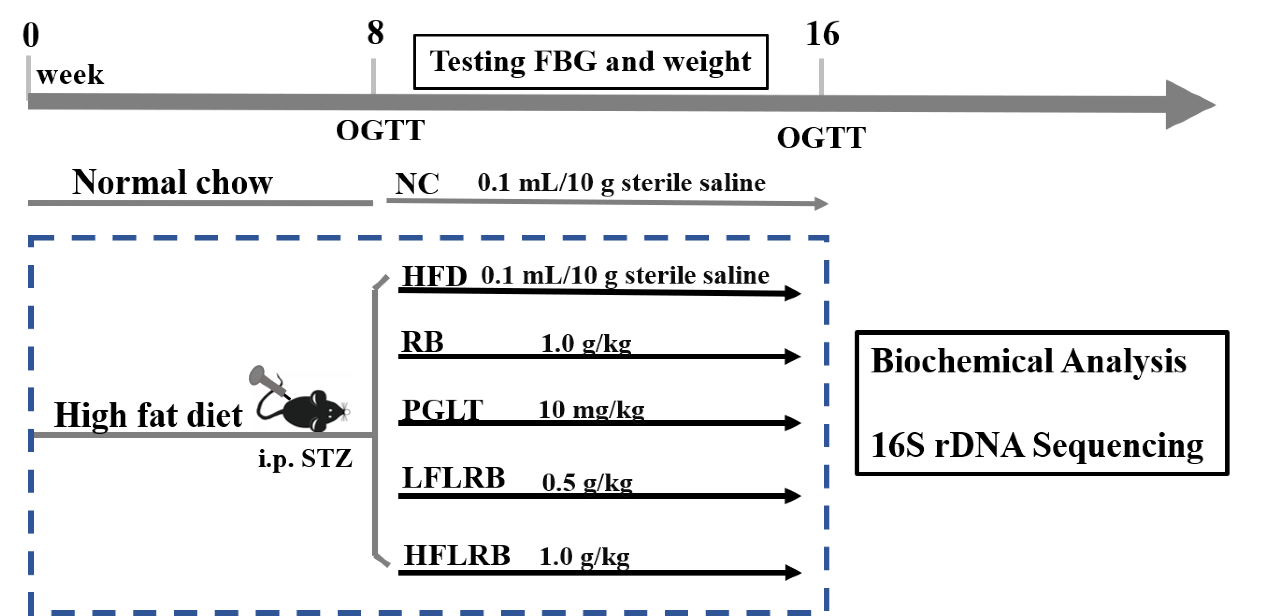


**Figure S2. Experiment schemes.**


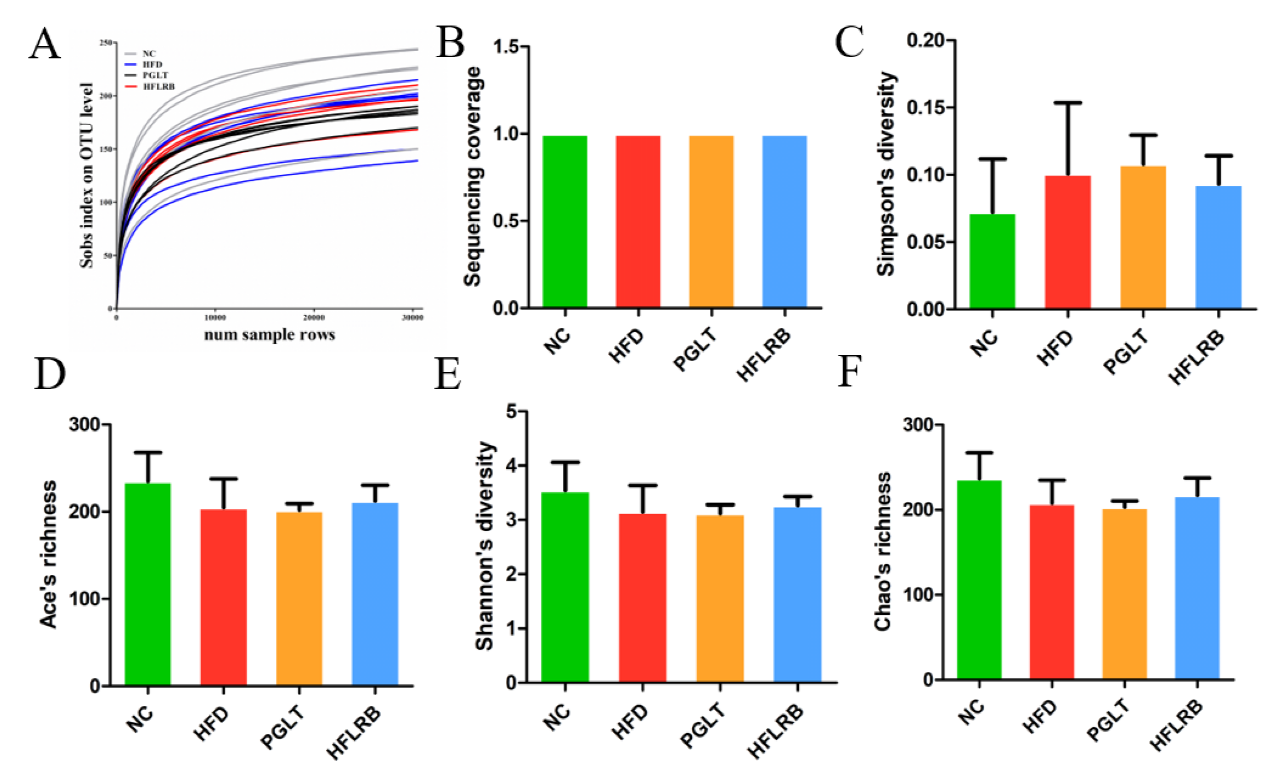


**Figure S3. Alpha-diversity of fecal samples collected from 24 mice.** (A) Rarefaction curve; (B) sequencing coverage; (C) Simpson diversity; (D) Ace richness; (E) Shannon diversity; (F) Chao richness.

**
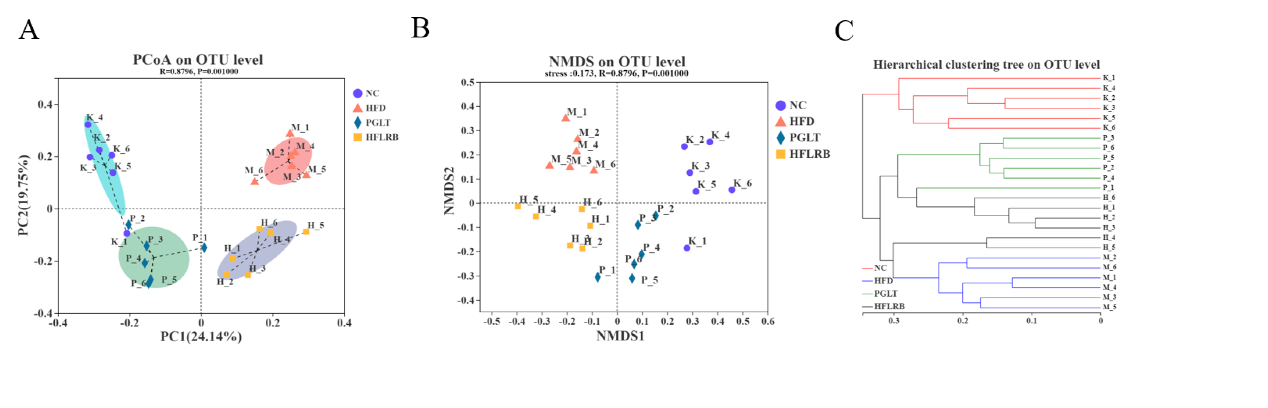
**

**Figure S4. Variations in microbial communities in samples from the NC, HFD, PGLT and HFLRB groups in diabetic mice.** (A) Principal coordinates analysis, (B) NMDS analysis of microbial community, (C) hierarchical clustering tree analysis.

**
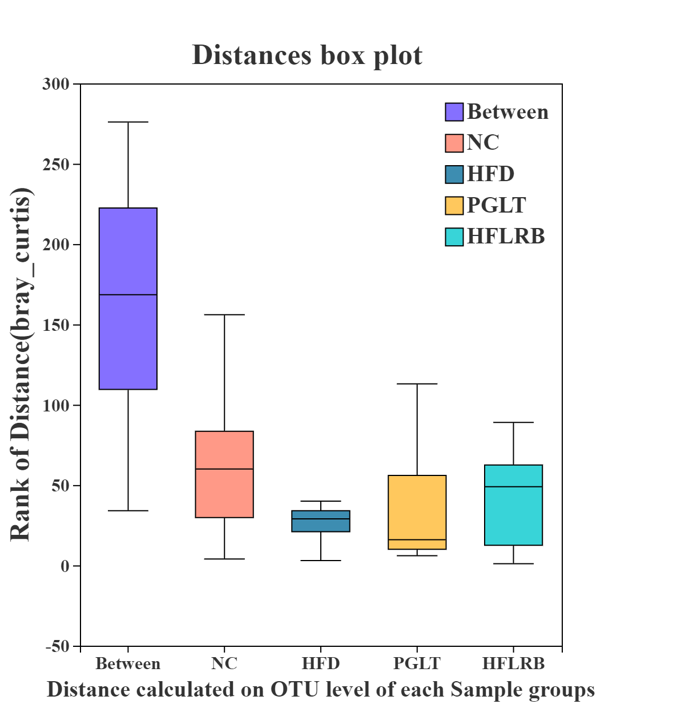
**

**Figure S5. Similarity analysis (AMOSIM) of gut microbiota in four groups of mice (NC, HFD, PGLT and HFLRB).**


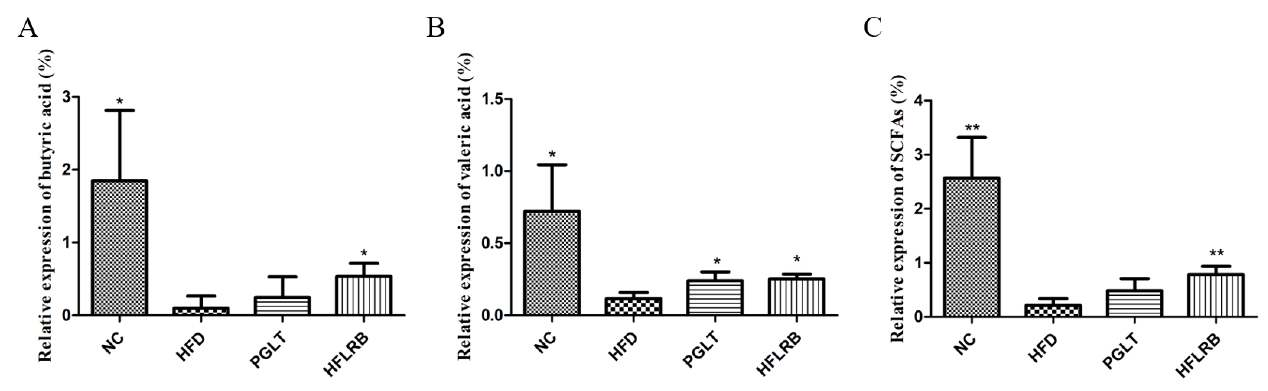


**Figure S6. Relative expression of SCFAs in fecal samples.** (A) Butyric acid, (B) valeric acid, (C) total SCFAs. ** represents *P*<0.01, * represents *P*<0.05, compared to the HFD.

**Table S1 Sequencing information statistics**

| **Amplified Region** | **Samples** | **Sequences** | **Bases(bp)** | **Average Length** |
| --- | --- | --- | --- | --- |
| 338F_806R | 24 | 1092405 | 4.57E+08 | 418.0634 |

**Table S2 Gut microbiota of 24 fecal samples at the phylum level**

| **Name** | **NC%** | **HFD%** | **PGLT%** | **HFLRB%** |
| --- | --- | --- | --- | --- |
| Firmicutes | 53.54 | 41.21 | 46.08 | 57.77 |
| Bacteroidota | 30.44 | 10.13 | 34.44 | 12.18 |
| Desulfobacterota | 1.73 | 10.32 | 7.31 | 16.2 |
| Verrucomicrobiota | 7.88 | 15.44 | 7.95 | 2.82 |
| Proteobacteria | 0.13 | 19.38 | 0.39 | 9.31 |
| Campilobacterota | 3.8 | 1.62 | 1.68 | 0.66 |
| Actinobacteriota | 1.98 | 1.66 | 0.22 | 0.27 |
| Deferribacterota | 0.14 | 0.25 | 1.93 | 0.79 |

**Table S3 Gut microbiota of 24 fecal samples at the genus level**

| **Name** | **HFD** | **HFLRB** | **NC** | **PGLT** |
| --- | --- | --- | --- | --- |
| Dubosiella | 1.085994 | 15.13064 | 9.539902 | 11.14155 |
| Lactobacillus | 0.773757 | 7.232849 | 1.933572 | 4.293667 |
| norank_f__Desulfovibrionaceae | 10.16492 | 16.0761 | 0.833361 | 7.163402 |
| norank_f__Muribaculaceae | 3.04636 | 5.674946 | 14.14198 | 28.74548 |
| Parabacteroides | 0.844844 | 2.647725 | 1.141223 | 0.587836 |
| Blautia | 1.753666 | 3.098308 | 2.161598 | 0.571979 |
| norank_f__Ruminococcaceae | 0.581275 | 1.606024 | 0.960224 | 0.664392 |
| Ruminococcus_torques_group | 0.518937 | 1.353391 | 0.060698 | 0.142721 |
| norank_f__Lachnospiraceae | 1.83569 | 2.507738 | 2.694205 | 1.333705 |
| Alloprevotella | 0.357076 | 0.536982 | 5.019303 | 1.437055 |
| unclassified_f__Lachnospiraceae | 8.436957 | 7.163949 | 5.139604 | 3.766528 |
| Clostridium_sensu_stricto_1 | 2.129882 | 0.101709 | 0.112099 | 0.026248 |
| Bacteroides | 4.749718 | 2.591949 | 6.830386 | 3.268917 |
| Faecalibaculum | 3.881361 | 1.112241 | 16.43755 | 4.240625 |
| Escherichia-Shigella | 17.43167 | 7.761081 | 0.039371 | 0.136706 |
| Akkermansia | 15.44069 | 2.815053 | 7.879742 | 7.949189 |
